# The characteristics of methicillin-resistant *Staphylococcus aureus* co-infection in COVID-19 pneumonia

**DOI:** 10.3389/fcimb.2025.1560688

**Published:** 2025-05-16

**Authors:** Feifei Gu, Yuyi Zhang, Jingyong Sun, Wei Guo, Lizhong Han

**Affiliations:** ^1^ Department of Laboratory Medicine, Ruijin Hospital, Shanghai Jiao Tong University School of Medicine, Shanghai, China; ^2^ Department of Laboratory Medicine, Zhongshan Hospital, Fudan University, Shanghai, China

**Keywords:** MRSA, *Staphylococcus aureus*, COVID-19, SARS-CoV-2, co-infection

## Abstract

Methicillin-resistant *Staphylococcus aureus* (MRSA) stands as a pervasive and important pathogen in the co-infections during the COVID-19 pandemic given its high morbidity and mortality. This study aimed to characterize MRSA isolates obtained from COVID-19 pneumonia with MRSA co-infection patients during the COVID-19 pandemic wave in China from 2022 to 2024. Fifty MRSA isolates collected form COVID-19 pneumonia with MRSA co-infection patients (MRSA-COC) and 50 MRSA isolates collected from MRSA pneumonia patients without COVID-19 (MRSA-CON) were enrolled in this study. Whole-genome sequencing, genomic epidemiology and comparative analysis were conducted to explore differences of MRSA isolates between two groups. Patients with MRSA-COC were significantly older (*P*=0.0492), had higher rates of severe pneumonia progression (*P*=0.0006), and carried greater comorbidity burdens. The time from hospital admission to MRSA detected was significantly shorter in MRSA-COC patients than in MRSA-CON patients (*P*=0.0141). Furthermore, MRSA-COC patients demonstrated significantly higher mortality rates compared with MRSA-CON patients (44% vs. 18%, *P*=0.0049). However, genomic analysis revealed no statistically significant differences in antimicrobial resistance gene or virulence factor genes between the MRSA-COC and MRSA-CON isolates. CC5 emerged as the predominant clone in both groups, with significantly higher prevalence in MRSA-COC isolates (88% vs. 66%, *P*=0.0090). The *tst*-positive ST5-MRSA-II strain was associated with concerning mortality rates in both MRSA-COC (50%) and MRSA-CON (20%) patients, underscoring the critical need for enhanced surveillance in MRSA pneumonia.

## Introduction

*Staphylococcus aureus* is an important and widespread bacterial pathogen, responsible for numerous uncomplicated skin infections and more severe, invasive infections worldwide each year. It is also a main causative agent of pneumonia leading to critical illness and death (He and Wunderink, 2020; Cheung et al., 2021). The pandemic coronavirus disease 2019 (COVID-19) is an infectious disease caused by the severe acute respiratory syndrome coronavirus 2 (SARS-CoV-2), has affected hundreds of thousands to millions of people resulting in a profound and detrimental effect on healthcare systems all over the world (Mirzaei et al., 2020). Co-infections and superinfections are common and frequently happened in respiratory viral infections, and secondary or bacterial co-infections with other viruses are known to substantially increase the mortality rate among viral-infected patients as previously documented (Mirzaei et al., 2020). Historically, *S. aureus* has been a major contributor to secondary bacterial infections in past viral pandemics and has significantly increased the mortality rates of infected patients (Cusumano et al., 2020). *S. aureus* co-infection and bacteremia were associated with nearly 50% mortality rates in patients during past influenza outbreaks, in contrast to the 1.4% morality rates in those infected with influenza alone (Leung et al., 2014).

There is a growing number of reports highlighting bacterial infections acquired by severe COVID-19 patients after hospital admission. In particular, nosocomial outbreaks in COVID-19 settings have been linked to methicillin-resistant *S. aureus* (MRSA), carbapenem-resistant *Acinetobacter baumannii*, and carbapenem-resistant *Klebsiella pneumoniae (* Segala et al., 2021). Antimicrobial-resistant pathogens including MRSA have been observed to cause healthcare-associated infections (HAIs) in COVID-19 patients, often leading to poorer clinical outcomes (O’Toole, 2021). A systematic review on the impact of COVID-19 pandemic on multidrug-resistant pathogens revealed significant changes in the rate of multidrug-resistant bacteria, and over half of the studies (54.5%) reported an increase in MRSA infection or colonization during the pandemic with the increase ranging from 4.6 to 170.6% (Abubakar et al., 2023). COVID-19 patients with *S. aureus* co-infection experience notably higher mortality rate during hospital admission compared with patients infected solely with COVID-19. *S. aureus* co-infection in COVID-19 patients is primarily associated with healthcare, and common hospital interventions such as intubation with mechanical ventilation, central venous catheter, and corticosteroids for severe COVID-19 patients heighten the risk of secondary bacterial infections (Adalbert et al., 2021).

China has experienced a dramatic COVID-19 wave since December 2022, with widespread primary SARS-CoV-2 infections across the population. A substantial proportion of these patients progressed to COVID-19 pneumonia requiring hospitalization, where bacterial co-infections were frequently observed and often associated with poor outcomes. Although the prevalence of COVID-19 in China has since stabilized at low levels, the large population and the extensive outbreak provided a unique context for studying secondary bacterial co-infections. To address the lack of research on MRSA co-infections in COVID-19 pneumonia, particularly in China, we conducted this study in hospitals across Shanghai, China. This study aims to shed light on the dynamics of MRSA co-infection in the context of COVID-19 pneumonia, filling a critical gap in the understanding of this clinical challenge.

## Materials and methods

### Study design

The patients diagnosed with MRSA co-infection following COVID-19 pneumonia from December 2022 to January 2024 in Ruijin hospital and Zhongshan hospital in Shanghai were enrolled in this study. For comparative analysis, MRSA pneumonia patients without COVID-19 during the same period were included as well. SARS-CoV-2 nucleic acid detection was used for COVID-19 diagnosis in Ruijin Hospital, and SARS-CoV-2 nucleic acid and/or antigen detections were used for COVID-19 diagnosis in Zhongshan Hospital. Ruijin Hospital (Shanghai Jiao Tong University School of Medicine) and Zhongshan Hospital (Fudan University) are both tertiary teaching hospitals in Shanghai, with bed capacities exceeding 3,000 and 2,000 beds respectively, providing comprehensive healthcare services to patients across China.

The diagnosis of community-acquired pneumonia (CAP) relies on the clinical symptoms of lower respiratory tract infection (e.g., dyspnea, productive cough, fever, and chills), combined with radiographic evidence of new pulmonary infiltrates and supportive laboratory findings (e.g., microbiological cultures, inflammatory markers, or molecular testing for pathogens) (Modi and Kovacs, 2020; Cilloniz et al., 2021; Torres et al., 2021). The symptoms of hospital-acquired pneumonia (HAP) may be hidden by either other medications or the cause of admission and HAP diagnosis is believed to be usually delayed (Torres et al., 2021), hence diagnosis of pneumonia can be complicated and requires comprehensive consideration. The pneumonia patients enrolled in this study were diagnosed by MRSA-positive sputum culture combined with clinical symptoms, as well as the presence of new infiltrates on chest radiographs, and/or other laboratory tests. The criteria for defining severe pneumonia comprises: (1) major criteria, including invasive mechanical ventilation, septic shock requiring vasopressor therapy; and (2) minor criteria, such as respiratory rate ≥30 breaths/min, PaO_2_/FiO_2_ ratio ≤250, multilobar infiltrates, confusion/disorientation, uremia (blood urea nitrogen level ≥20 mg/dL), leukopenia (white blood cell count <4 ×10^9^/L), thrombocytopenia (platelet count <100×10^9^/L), hypothermia (core temperature <36°C), hypotension requiring aggressive fluid resuscitation (Pletz et al., 2020; Budinger et al., 2021; Cilloniz et al., 2021).

### MRSA isolates

A total of 100 MRSA isolates were collected from sputum samples of pneumonia patients and included in this study. Among the 100 MRSA isolates, 50 MRSA isolates were collected from MRSA co-infection with COVID-19 pneumonia patients and named as group MRSA-COC (MRSA co-infection with COVID-19), and the other 50 MRSA isolates were collected from MRSA pneumonia patients without COVID-19 and named as group MRSA-CON (MRSA pneumonia but COVID-19 negative). Group MRSA-COC included 27 MRSA isolates from Ruijin Hospital and 23 MRSA isolates from Zhongshan Hospital. Fifty MRSA isolates of group MRSA-CON (27 from Ruijin Hospital and 23 from Zhongshan Hospital) were randomly selected using the random number generation function of Microsoft Excel 365 (Microsoft Corporation, Redmond, WA, USA).

Ethical approval for this retrospective study was granted by the Ethics Committee of Ruijin Hospital, Shanghai Jiao Tong University School of Medicine. The requirement for informed consent was waived, as the research focused solely on bacterial analysis without involving patient interventions.

### Identification and MRSA confirmation

Initial species identification of isolates was conducted using MALDI-TOF mass spectrometry (bioMérieux, Marcy-l’Étoile, France). MRSA was screened by the cefoxitin (30 μg) disk diffusion method following the Clinical and Laboratory Standards Institute guidelines (Institute CaLS, 2023). The presence of the *mecA* was confirmed to validate MRSA identification.

### Genome sequencing and assembly

Genomic DNA was extracted using the Wizard^®^ Genomic DNA Purification Kit (Promega). DNA samples were fragmented into 400–500 bp segments using the Covaris M220 Focused Acoustic Shearer, following the manufacturer’s protocol. Illumina sequencing libraries were prepared from the sheared fragments with the NEXTflex™ Rapid DNA-Seq Kit. Raw sequencing reads were processed using fastp software (version 0.19.6) for quality filtering and subsequently assembled using the SOPA *de novo* assembler (version 2.04) (Luo et al., 2012).

### Genomic epidemiology analysis

The assembled contigs were analyzed for genomic epidemiology using the Center for Genomic Epidemiology (CGE) platform (http://www.genomicepidemiology.org/). Analyses included phenotyping (antibiotic resistance genes and virulence factor genes) and typing (Multilocus sequence typing [MLST], *spa* type, and SCC*mec* type).

### Statistical analysis

Statistical analysis was conducted using the t-test, chi-square test, or Fisher’s exact test, as appropriate. A two-sided *P* value of < 0.05 was considered statistically significant. All analyses were performed using SAS 8.2 software (SAS Institute Inc., Cary, NC, USA).

## Results

### Clinical data

From December 2022 to January 2024, 100 pneumonia patients (54 from Ruijin Hospital, 46 from Zhongshan Hospital) were enrolled in this study. Among them, 50 patients were diagnosed with MRSA co-infection with COVID-19 pneumonia (MRSA-COC), and the other 50 patients were diagnosed with MRSA pneumonia without COVID-19 (MRSA-CON). The median time from COVID-19 (SARS-CoV-2) positive-tested to MRSA detected was 10.5 days (range: 0–69 days). The median time from hospital admission to MRSA detected was 13.5 days (range: 1–367 days) and 22.5 days (range: 1–732 days) respectively in the MRSA-COC and MRSA-CON patients as shown in **Table 1**, and the time from admission to MRSA detected was significantly shorter in the MRSA-COC patients compared with MRSA-CON patients (*P*=0.0141). The age of patients with MRSA-COC was higher than those with MRSA-CON (*P*=0.0492). The patients with MRSA-COC demonstrated significantly higher rates of severe pneumonia progression (72% vs. 38%, *P*=0.0006) and greater comorbidity burdens such as cardiac diseases (64% vs. 38%, *P*=0.0093), hypertension (68% vs. 28%, *P*<0.0001), diabetes mellitus (46% vs. 24%, *P*=0.0211) and kidney diseases (32% vs. 14%, *P*=0.0325). Conversely, the patients with MRSA-CON were more frequently associated with underlying respiratory conditions (14% vs. 32%, *P*=0.0325). The mortality rate was significantly higher in MRSA-COC patients compared with MRSA-CON patients (44% vs. 18%, *P*=0.0049).

**Table 1:** Clinical data of MRSA pneumonia patients with/without COVID-19.

Clinical data	Total (n=100)	MRSA-COC Patients (n=50)	MRSA-CON Patients (n=50)	*P* value
Demographic characteristic				
Age, median (range)	75 (19-98)	77.5 (36-98)	73 (19-95)	0.0492
Male sex	77	37	40	0.4759
Hospitalization				
Time from admission to MRSA detected, median days (range)	15.5(1-732)	13.5(1-367)	22.5(1-732)	0.0141
Diagnosis and comorbidities				
Severe pneumonia	55	36	19	0.0006
Cardiac diseases	51	32	19	0.0093
Brain diseases	38	15	23	0.0993
Hypertension	58	34	14	< 0.0001
Diabetes mellitus	35	23	12	0.0211
Liver diseases	21	9	12	0.4614
Kidney diseases	23	16	7	0.0325
Cancer	14	6	8	0.5644
Mental disorder	18	7	11	0.2978
Parkinson	7	4	3	1.0000
Neurological diseases	9	3	6	0.4846
Respiratory diseases	23	7	16	0.0325
Hematological diseases	24	11	13	0.6396
Digestive diseases	20	8	12	0.3173
ECMO use	2	1	1	1.0000
Tracheotomy	22	14	8	0.1475
Tracheal intubation	30	18	12	0.1904
Ventilator use	34	13	21	0.0913
Outcomes				
Death	31	22	9	0.0049

### Antimicrobial resistance genes

No genes or mutations associated with resistance to oxazolidinones, glycopeptides or lipoglycopeptides were identified in this study. Aminoglycosides resistance genes including *aac(6’)-aph(2’’), aph(2’’)-Ia, aph(3’)-III, aadD* were detected in 71 isolates. Genes conferring resistance to MLS-B (macrolides, lincosamides, ketolides, and streptogramin B) such as *ermA*, *ermB* or *ermC* were found in 84 isolates. Tetracycline resistance genes *tetM* and/or *tetK* were detected in 80 isolates. High-level mupirocin resistance gene *mupA* was present in 50 isolates. No significant differences in antimicrobial resistance genes were observed between MRSA-COC and MRSA-CON groups as presented in **Table 2**.

**Table 2:** The antimicrobial resistance genes of MRSA isolates from MRSA pneumonia patients with/without COVID-19.

Antimicrobial	Antimicrobial resistance genes	Total (n=100)	MRSA-COC (n=50)	MRSA-CON (n=50)	*P* value
Aminoglycosides	*aac(6’)-aph(2’’)*	59	30	29	0.8389
	*aph(2’’)-Ia*	2	2	0	0.4751
	*aph(3’)-III*	4	4	0	0.1258
	*aadD*	11	4	7	0.3377
Spectinomycin	*ant(9)-Ia*	77	40	37	0.4759
	*ant(6)-Ia*	3	3	0	0.2410
Beta-lactamase	*blaZ*	23	11	12	0.8122
Fosfomycin	*fosY*	8	4	4	1.0000
	*fosB*	5	2	3	1.0000
	*fosD*	1	0	1	1.0000
Lincomycin	*lnu(G)*	8	4	4	1.0000
	*lnu(A)*	4	1	3	0.6098
MLS-B	*erm(A)*	76	39	37	0.6396
	*erm(B)*	5	3	2	1.0000
	*erm(C)*	5	3	2	1.0000
Tetracycline	*tet(M)*	74	39	35	0.3618
	*tet(K)*	39	18	21	0.5385
Bleomycin	*bleO*	5	2	3	1.0000
Mupirocin	*mupA*	50	25	25	1.0000
Chloramphenicol	*fexA*	1	1	0	1.0000
	*cat*	2	1	1	1.0000
Trimethoprim	*dfrE*	2	2	0	0.4751

MLS-B, macrolides, lincosamides, ketolides, and streptogramin B.

### Virulence factor genes

All MRSA isolates in this study carried genes encoding aureolysin (*aur*) and hemolysin (*hlgA, hlgB, hlgC*), as detailed in **Table 3**. The *tst* gene (encoding toxic shock syndrome toxin-1) was identified in 13 isolates overall (8 from MRSA-COC group and 5 from MRSA-CON group). The gene *lukS/F-PV* encoding Panton-Valentine leukocidin was found in only one MRSA isolate in the MRSA-COC group. No significant differences in the distribution of virulence factor genes were observed between two groups as shown in **Table 3**.

**Table 3:** The virulence factor genes of MRSA isolates from MRSA pneumonia patients with/without COVID-19.

Virulence factor genes	Total (n=100)	MRSA-COC (n=50)	MRSA-CON (n=50)	*P* value
Hostimm genes				
*sak*	84	41	43	0.5854
*scn*	82	39	43	0.2978
Exoenzyme genes				
*aur*	100	50	50	–
*splA*	88	46	42	0.2184
*splB*	88	46	42	0.2184
*splE*	8	4	4	1.0000
Toxin genes				
*hlgA*	100	50	50	–
*hlgB*	100	50	50	–
*hlgC*	100	50	50	–
*lukS/F-PV*	1	1	0	1.0000
*lukD*	88	46	42	0.2184
*lukE*	88	46	42	0.2184
*sea*	17	9	8	0.7901
*seb*	64	31	33	0.6769
*sec*	21	12	9	0.4614
*seg*	78	40	38	0.6292
*seh*	8	4	4	1.0000
*sei*	78	40	38	0.6292
*sek*	13	6	7	0.7662
*sel*	19	10	9	0.7988
*sem*	78	40	38	0.6292
*sen*	78	40	38	0.6292
*seo*	78	40	38	0.6292
*seq*	13	6	7	0.7662
*seu*	78	40	38	0.6292
*tst*	13	8	5	0.3724

### Molecular types

As presented in **Table 4**, the clonal complex CC5 was the most prevalent (77/100, 77%), found in 88% (44/50) of MRSA-COC isolates and 66% (33/50) of MRSA-CON isolates, and CC5 was much more frequently found in MRSA-COC group (88% vs. 66%, *P*=0.0090). All 13 *tst*-positive isolates exclusively comprised ST5-MRSA (CC5), with mortality rates of 50% (4/8) in MRSA-COC patients and 20% (1/5) in MRSA-CON patients. Overall, the most common sequence type (ST) and *spa* type were ST764 (62/100, 62%) and t002 (56/100, 56%) respectively. SCC*mec*II (75%) was the most frequent SCC*mec* type, followed by SCC*mec*IV (14%), SCC*mec*V (9%), and SCC*mec*III (2%). Among MRSA-COC isolates, ST764 (30/50, 60%), t002 (30/50, 60%), and SCC*mec*II (38/50, 76%) were the most common molecular types.

**Table 4:** The molecular types of MRSA isolates from MRSA pneumonia patients with/without COVID-19.

CC (n)	ST (n)	SCC*mec* type (n)	*spa* type (n)	Virulence factor genes (n)	MRSA-COC (n=50)	MRSA-CON (n=50)	*P* value
5 (77)	ST5 (13)	II (13)	t2460 (7), t311 (2), t5076 (2), t002 (1), t5067 (1)	*sak* (5), *scn* (3), *aur* (13), *splA* (13), *splB* (13), *hlgA* (13), *hlgB* (13), *hlgC* (13), *lukD* (13), *lukE* (13), *sea* (2), *sec* (13), *seg* (13), *sei* (13), *sel* (11), *sem* (13), *sen* (13), *seo* (13), *seu* (13), *tst* (13)	8	5	0.3724
	ST764 (62)	II (62)	t002 (54), t045 (2), t311 (2), t509 (2), t1084 (1), t447 (1)	*sak* (57), *scn* (57), *aur* (62), *splA* (62), *splB* (62), *hlgA* (62), *hlgB* (62), *hlgC* (62), *lukD* (62), *lukE* (62), *seb* (58), *seg* (62), *sei* (62), *sem* (62), *sen* (62), *seo* (62), *seu* (62)	34	28	0.2164
	ST965 (2)	IVc (2)	t2049 (1), t653 (1)	*sak* (2), *scn* (2), *aur* (2), *splA* (2), *splB* (2), *hlgA* (2), *hlgB* (2), *hlgC* (2), *lukD* (2), *lukE* (2), *sea* (2), *seg* (2), *sei* (2), *sem* (2), *sen* (2), *seo* (2), *seu* (2)	2	0	0.4751
1 (8)	ST1 (8)	IVc (7)	t127 (5), t002 (1), t321 (1)	*sak* (7), *scn* (7), *aur* (7), *splA* (7), *splB* (7), *splE* (7), *hlgA* (7), *hlgB* (7), *hlgC* (7), *lukD* (7), *lukE* (7), *sea* (7), *sec* (7), *seh* (7), *sek* (7), *sel* (7), *seq* (7)	4	3	1.0000
		IV (1)	t127 (1)	*sak* (1), *scn* (1), *aur* (1), *splA* (1), *splB* (1), *splE* (1), *hlgA* (1), *hlgB* (1), *hlgC* (1), *lukD* (1), *lukE* (1), *sea* (1), *sec* (1), *seh* (1), *sek* (1), *sel* (1), *seq* (1)	0	1	1.0000
398 (8)	ST398 (8)	V (8)	t034 (6), t19605 (1), new (1)	*sak* (6), *scn* (5), *aur* (8), *splA* (1), *splB* (1), *hlgA* (8), *hlgB* (8), *hlgC* (8), *lukD* (1), *lukE* (1), *seb* (1), *seg* (1), *sei* (1), *sem* (1), *sen* (1), *seo* (1), *seu* (1)	2	6	0.2688
59 (5)	ST59 (5)	IVa (4)	t437 (2), t163 (1), t172(1)	*sak* (4), *scn* (4), *aur* (4), *hlgA* (4), *hlgB* (4), *hlgC* (4), *sea* (3), *seb* (4), *sek* (4), *seq* (4)	1	3	0.6098
		Vb (1)	t441 (1)	*scn* (1), *aur* (1), *hlgA* (1), *hlgB* (1), *hlgC* (1), *lukS/F-PV* (1), *seb* (1), *sek* (1), *seq* (1)	1	0	1.0000
8 (2)	ST239 (2)	III (2)	t030 (2)	*sak* (2), *scn* (2), *aur* (2), *splA* (2), *splB* (2), *hlgA* (2), *hlgB* (2), *hlgC* (2), *lukD* (2), *lukE* (2), *sea* (2)	2	0	0.4751

## Discussion

COVID-19, characterized by high transmission rates, poses severe complications including acute respiratory distress syndrome (ARDS), thromboembolic events, septic shock, and multi-organ failure. Bacterial co-infection in COVID-19 patients often exacerbates the immunosuppressive effects of the viral infection, worsening clinical outcomes (Adalbert et al., 2021). According to the results we studied, the mortality rate among the COVID-19 pneumonia patients with MRSA co-infection was significantly higher than that among the MRSA pneumonia patients without COVID-19 (44% vs. 18%, *P*=0.0049). This mortality rate was also much higher than previously we have studied for *S. aureus* bloodstream infections in Shanghai in recent years (Gu et al., 2020). These findings align with previous reviews, which demonstrate that *S. aureus* co-infection in COVID-19 pneumonia patients leads to worse outcomes compared with those with COVID-19 or MRSA solely during hospital admission (Adalbert et al., 2021; O’Toole, 2021; Abubakar et al., 2023). MRSA co-infection in COVID-19 patients is predominantly associated with healthcare, and common hospital interventions for severe COVID-19 patients, such as prolonged hospitalization and invasive procedures, may increase the risk of bacterial co-infection. To mitigate the risks, preventive measures such as COVID-19 vaccination and outpatient care may be key initiatives to reduce hospital admissions and subsequent MRSA co-infections. Infection prevention, control programs, and antimicrobial stewardship must be prioritized, particularly in light of the long-term burden of HAIs during the COVID- 19 pandemic (O’Toole, 2021).

Although no significant differences in antimicrobial resistance profiles were found between MRSA-COC and MRSA-CON isolates, antimicrobial resistance (AMR) problem remains a critical concern in MRSA co-infection in COVID-19. Many antibiotics such as azithromycin have been used to prevent and treat bacterial co-infection and secondary bacterial infections in patients with viral respiratory infections such as SARS-CoV-2. Although antibiotics do not directly affect SARS-CoV-2, viral respiratory infections often lead to bacterial pneumonia (Mirzaei et al., 2020). The high prevalence of MLS-B antibiotics resistance observed in this study (overall: 84/100, 84%; MRSA-COC isolates: 43/50, 86%; MRSA-CON isolates: 41/50, 82%) may significantly compromise therapeutic efficacy in both MRSA co-infected COVID-19 pneumonia and MRSA pneumonia cases. The overuse of broad-spectrum antibiotics during the COVID-19 pandemic may have inadvertently accelerated the emergence of antimicrobial-resistant pathogens, emphasizing the need for robust surveillance and antimicrobial stewardship programs. As demonstrated by our findings, no resistance to oxazolidinones, glycopeptides or lipoglycopeptides was detected, suggesting these antibiotic classes may remain viable for MRSA pneumonia treatment regardless of COVID-19 infection status. However, continuous vigilance against emerging resistant strains is deeply imperative.

In acute and intensive care units, inappropriate antimicrobial exposure and disrupted infection control measures have contributed to the selection and spread of antimicrobial-resistant pathogens. The COVID-19 pandemic is likely to play an important role in the emergence and transmission of resistant pathogens in hospital settings, hence there is a need to enhance infection prevention and antimicrobial management, as well as robust and consistent surveillance for antimicrobial resistance as part of the pandemic response and recovery (Langford et al., 2023). Antimicrobial resistance will continue to pose a substantial threat to healthcare systems in the coming years. To mitigate the potential long-term impact of COVID-19 on antimicrobial resistance, it is necessary to integrate both infection prevention and control strategies and antimicrobial stewardship activities into the pandemic response. Management strategies to reduce the emergence of AMR should therefore be investigated and implemented at local and global levels (Rehman, 2023). Proper prescription and optimized use of antimicrobials, coupled with high-quality diagnostics and aggressive infection control measures, may prevent the onset of multidrug-resistant pathogens during the pandemic (Lai et al., 2021). Research into new therapeutic approaches and improved diagnostic tools can help reduce reliance on broad-spectrum antibiotics and prevent the diffusion of multidrug-resistant pathogens.

Consistent with the findings of antimicrobial resistance genes in this study, no statistically significant differences in virulence factor genes were observed between MRSA-COC and MRSA-CON groups. These findings suggest that MRSA strains exhibit comparable levels of antimicrobial resistance and pathogenicity in both MRSA co-infection with COVID-19 pneumonia and MRSA pneumonia cases, warranting equal clinical attention. Consequently, similar antibiotic treatment and prevention strategies may be adopted for MRSA pneumonia patients with/without COVID-19. However, the higher mortality rate observed in MRSA co-infected COVID-19 pneumonia cases compared with MRSA pneumonia cases may be attributed to the underlying comorbidities and COVID-19-related pathological effects, which require specific clinical interventions and targeted therapies.

CC5 is one of the five major global MRSA clonal complexes (CC5, CC8, CC22, CC30, CC45) and has been frequently reported worldwide including in the USA, South America, Europe, Asia, Africa, and Australia (Lakhundi and Zhang, 2018). In this study, CC5 was the dominant clonal complex, present in 77% of MRSA isolates, with significantly higher prevalence in MRSA-COC group than in MRSA-CON isolates (88% vs. 66%, *P*=0.0090). This aligns with reports identifying CC5 as a major global MRSA clonal complex associated with endemic outbreaks in regions with high MRSA morbidity rates (Wang et al., 2022). As presented in **Table 4** in our current study, it was revealed that all 13 MRSA isolates carrying the *tst* gene pertained to the ST5-MRSA-II lineage. Notably, this *tst*-positive ST5-MRSA-II strain exhibited high mortality rates in both MRSA-COC patients (50%) and MRSA-CON patients (20%). The close binding-like relationship between *tst* gene and CC5 (ST5) has been discovered in our precious studies about *S. aureus* bloodstream infections and MRSA burn wound infection (Gu et al., 2020; Gu et al., 2023). The toxin gene *tst* encodes Toxic Shock Syndrome Toxin-1 (TSST-1) which is a 22 kDa extracellular protein toxin secreted by *S. aureus*, a virulence factor that triggers immune dysregulation, fever, rash, vascular disorders, toxic shock syndrome vascular disorders, and multi-organ failure (Zhu et al., 2023). The TSST-1 gene (*tst*) has also been found predominantly in CC5 MRSA isolates in Suzhou, China associated with higher mortality (Wang et al., 2017) and *tst*-positive ST5-MRSA-II has been proved to be represented one dominated clone in China as reported (Zhao et al., 2019). These findings highlight the need for strict infection prevention and control measures and innovative therapeutic strategies to prevent further spread of the *tst*-positive CC5 MRSA strains in China, particularly in Shanghai and Zhejiang province. Some protein synthesis inhibitory antibiotics targeting TSST-1 or herbal extracts inhibiting the production of the TSST-1 toxin can be considered as an potential alternative strategy for managing *tst*-positive *S. aureus* infection because toxic shock syndrome (TSS) caused by TSST-1 is frequently fatal without prompt and targeted therapeutic intervention (Qiu et al., 2011; Hodille et al., 2017; Katahira et al., 2019). In addition, it has been discovered that the expression of *tst* potentially associated with the mutation of its promoter and variations in specific virulence regulators expression (Zhao et al., 2019). Therefore, sequencing of the *tst* promoter and quantitative analysis of major virulence regulators expression may provide important information for investigation of MRSA infection.

Given the close association between CC5-MRSA and key virulence factors such as *tst* as reported, enhanced global and regional surveillance of CC5-MRSA lineage is critically needed. Further genetic investigations into the *tst* promoter region and the regulatory mechanisms of virulence factor expression may yield insights into its pathogenesis and resistance. Implementing integrated infection control strategies alongside antimicrobial stewardship programs is vital to curbing the dissemination of hypervirulent and highly transmissible CC5-MRSA strains. Additionally, advancing diagnostic precision and developing targeted therapeutic approaches are imperative to mitigate the escalating threat of antimicrobial resistance and optimize clinical outcomes.

This study is limited by the inclusion of only two hospitals in Shanghai, which may affect its generalizability and introduce potential bias. The findings should therefore be interpreted with caution, as they represent only a subset of MRSA pneumonia patients in Shanghai. Future multicenter studies incorporating a broader range of hospitals are needed to enhance epidemiological representativeness and yield more robust findings.

In conclusion, this study revealed that the patients with MRSA-COC were significantly older, exhibited higher rates of severe pneumonia progression with greater comorbidity burdens, and showed shorter time from admission to MRSA infection compared with MRSA-CON patients. The mortality rate of MRSA-COC patients was significantly higher than that of MRSA-CON patients. However, genomic analysis revealed no significant differences in antimicrobial resistance genes or virulence factors genes between two groups, suggesting comparable antimicrobial resistant and pathogenic potential between MRSA strains causing MRSA co-infections in COVID-19 pneumonia and conventional MRSA pneumonia. Clonal distribution analysis identified CC5 as the dominant clone in both groups, with significantly higher prevalence among MRSA-COC isolates. Most critically, *tst*-positive ST5-MRSA-II strains demonstrated concerning mortality rates in both MRSA-COC and MRSA-CON pneumonia patients, highlighting the urgent need for enhanced surveillance and tailored management strategies for MRSA pneumonia especially in COVID-19 pneumonia.

## Data Availability

The original contributions presented in the study are included in the article/supplementary material. Further inquiries can be directed to the corresponding authors.

## References

[B1] Abubakar U. Al-Anazi M. Alanazi Z. Rodriguez-Bano J. (2023). Impact of COVID-19 pandemic on multidrug resistant gram positive and gram negative pathogens: A systematic review. J. Infect. Public Health 16, 320–331. doi: 10.1016/j.jiph.2022.12.022 36657243 PMC9804969

[B2] Adalbert J. R. Varshney K. Tobin R. Pajaro R. (2021). Clinical outcomes in patients co-infected with COVID-19 and *Staphylococcus aureus*: a scoping review. BMC Infect. Dis. 21, 985. doi: 10.1186/s12879-021-06616-4 34548027 PMC8453255

[B3] Budinger G. R. S. Misharin A. V. Ridge K. M. Singer B. D. Wunderink R. G. (2021). Distinctive features of severe SARS-CoV-2 pneumonia. J. Clin. Invest. 131, e149412. doi: 10.1172/JCI149412 34263736 PMC8279580

[B4] Cheung G. Y. C. Bae J. S. Otto M. (2021). Pathogenicity and virulence of Staphylococcus aureus. Virulence 12, 547–569. doi: 10.1080/21505594.2021.1878688 33522395 PMC7872022

[B5] Cilloniz C. Torres A. Niederman M. S. (2021). Management of pneumonia in critically ill patients. BMJ 375, e065871. doi: 10.1136/bmj-2021-065871 34872910

[B6] Cusumano J. A. Dupper A. C. Malik Y. Gavioli E. M. Banga J. Berbel Caban A.. (2020). *Staphylococcus aureus* bacteremia in patients infected with COVID-19: A case series. Open Forum Infect. Dis. 7, ofaa518. doi: 10.1093/ofid/ofaa518 33269299 PMC7686656

[B7] Gu F. He W. Xiao S. Wang S. Li X. Zeng Q.. (2020). Antimicrobial Resistance and Molecular Epidemiology of *Staphylococcus aureus* Causing Bloodstream Infections at Ruijin Hospital in Shanghai from 2013 to 2018. Sci. Rep. 10, 6019. doi: 10.1038/s41598-020-63248-5 32265473 PMC7138830

[B8] Gu F. He W. Zhu D. Yang P. Sun J. Han L. (2023). A 10-year retrospective study of methicillin-resistant *Staphylococcus aureus* from burn wound infection in southeast China from 2013 to 2022. Front. Microbiol. 14. doi: 10.3389/fmicb.2023.1301744 PMC10722408 38107851

[B9] He H. Wunderink R. G. (2020). *Staphylococcus aureus* pneumonia in the community. Semin. respiratory Crit. Care Med. 41, 470–479. doi: 10.1055/s-0040-1709992 32521547

[B10] Hodille E. Rose W. Diep B. A. Goutelle S. Lina G. Dumitrescu O. (2017). The role of antibiotics in modulating virulence in staphylococcus aureus. Clin. Microbiol. Rev. 30, 887–917. doi: 10.1128/cmr.00120-16 28724662 PMC5608880

[B11] Institute CaLS (2023). Performance Standards for Antimicrobial Susceptibility Testing Vol. 43 (Wayne, PA, USA: CLSI document M100-S29).

[B12] Katahira E. J. Davidson S. M. Stevens D. L. Bolz D. D. (2019). Subinhibitory concentrations of tedizolid potently inhibit extracellular toxin production by methicillin-sensitive and methicillin-resistant Staphylococcus aureus. J. Med. Microbiol. 68, 255–262. doi: 10.1099/jmm.0.000905 30556803 PMC6557147

[B13] Lai C. C. Chen S. Y. Ko W. C. Hsueh P. R. (2021). Increased antimicrobial resistance during the COVID-19 pandemic. Int. J. Antimicrob. Agents 57, 106324. doi: 10.1016/j.ijantimicag.2021.106324 33746045 PMC7972869

[B14] Lakhundi S. Zhang K. (2018). Methicillin-resistant *staphylococcus aureus*: molecular characterization, evolution, and epidemiology. Clin. Microbiol. Rev. 31, e00020–e00018. doi: 10.1128/CMR.00020-18 30209034 PMC6148192

[B15] Langford B. J. Soucy J. R. Leung V. So M. Kwan A. T. H. Portnoff J. S.. (2023). Antibiotic resistance associated with the COVID-19 pandemic: a systematic review and meta-analysis. Clin. Microbiol. Infect. 29, 302–309. doi: 10.1016/j.cmi.2022.12.006 36509377 PMC9733301

[B16] Leung C. H. Tseng H. K. Wang W. S. Chiang H. T. Wu A. Y. Liu C. P. (2014). Clinical characteristics of children and adults hospitalized for influenza virus infection. J. microbiol. immunol. infection = Wei mian yu gan ran za zhi 47, 518–525. doi: 10.1016/j.jmii.2013.06.002 23932366

[B17] Luo R. Liu B. Xie Y. Li Z. Huang W. Yuan J.. (2012). SOAPdenovo2: an empirically improved memory-efficient short-read *de novo* assembler. Gigascience 1, 18. doi: 10.1186/2047-217x-1-18 23587118 PMC3626529

[B18] Mirzaei R. Goodarzi P. Asadi M. Soltani A. Aljanabi H. A. A. Jeda A. S.. (2020). Bacterial co-infections with SARS-coV-2. IUBMB Life 72, 2097–2111. doi: 10.1002/iub.2356 32770825 PMC7436231

[B19] Modi A. R. Kovacs C. S. (2020). Hospital-acquired and ventilator-associated pneumonia: Diagnosis, management, and prevention. Cleve Clin. J. Med. 87, 633–639. doi: 10.3949/ccjm.87a.19117 33004324

[B20] O’Toole R. F. (2021). The interface between COVID-19 and bacterial healthcare-associated infections. Clin. Microbiol. Infect. 27, 1772–1776. doi: 10.1016/j.cmi.2021.06.001 34111586 PMC8182977

[B21] Pletz M. W. Blasi F. Chalmers J. D. Dela Cruz C. S. Feldman C. Luna C. M.. (2020). International perspective on the new 2019 american thoracic society/infectious diseases society of america community-acquired pneumonia guideline: A critical appraisal by a global expert panel. Chest 158, 1912–1918. doi: 10.1016/j.chest.2020.07.089 32858009 PMC7445464

[B22] Qiu J. Wang J. Luo H. Du X. Li H. Luo M.. (2011). The effects of subinhibitory concentrations of costus oil on virulence factor production in Staphylococcus aureus. J. Appl. Microbiol. 110, 333–340. doi: 10.1111/j.1365-2672.2010.04888.x 21070517

[B23] Rehman S. (2023). A parallel and silent emerging pandemic: Antimicrobial resistance (AMR) amid COVID-19 pandemic. J. Infect. Public Health 16, 611–617. doi: 10.1016/j.jiph.2023.02.021 36857834 PMC9942450

[B24] Segala F. V. Bavaro D. F. Di Gennaro F. Salvati F. Marotta C. Saracino A.. (2021). Impact of SARS-coV-2 epidemic on antimicrobial resistance: A literature review. Viruses 13, 2110. doi: 10.3390/v13112110 34834917 PMC8624326

[B25] Torres A. Cilloniz C. Niederman M. S. Menéndez R. Chalmers J. D. Wunderink R. G.. (2021). Pneumonia. Nat. Rev. Dis. Primers 7, 25. doi: 10.1038/s41572-021-00259-0 33833230

[B26] Wang B. Xu Y. Zhao H. Wang X. Rao L. Guo Y.. (2022). Methicillin-resistant *Staphylococcus aureus* in China: a multicentre longitudinal study and whole-genome sequencing. Emerging microbes infections 11, 532–542. doi: 10.1080/22221751.2022.2032373 35060838 PMC8843102

[B27] Wang M. Zheng Y. Mediavilla J. R. Chen L. Kreiswirth B. N. Song Y.. (2017). Hospital Dissemination of tst-1-Positive Clonal Complex 5 (CC5) Methicillin-Resistant Staphylococcus aureus. Front. Cell. infection Microbiol. 7. doi: 10.3389/fcimb.2017.00101 PMC5374150 28409124

[B28] Zhao H. Xu S. Yang H. He C. Xu X. Hu F.. (2019). Molecular Typing and Variations in Amount of tst Gene Expression of TSST-1-Producing Clinical *Staphylococcus aureus* Isolates. Front. Microbiol. 10. doi: 10.3389/fmicb.2019.01388 PMC6594356 31275293

[B29] Zhu Z. Hu Z. Li S. Fang R. Ono H. K. Hu D.-L. (2023). Molecular characteristics and pathogenicity of *staphylococcus aureus* exotoxins. Int. J. Mol. Sci. 25, 395. doi: 10.3390/ijms25010395 38203566 PMC10778951

